# Toll-like receptors 2, 4, and 9 modulate promoting effect of COPD-like airway inflammation on K-ras-driven lung cancer through activation of the MyD88/NF-ĸB pathway in the airway epithelium

**DOI:** 10.3389/fimmu.2023.1118721

**Published:** 2023-05-22

**Authors:** Walter V. Velasco, Nasim Khosravi, Susana Castro-Pando, Nelly Torres-Garza, Maria T. Grimaldo, Avantika Krishna, Michael J. Clowers, Misha Umer, Sabah Tariq Amir, Diana Del Bosque, Soudabeh Daliri, Maria Miguelina De La Garza, Marco Ramos-Castaneda, Scott E. Evans, Seyed Javad Moghaddam

**Affiliations:** ^1^ Department of Pulmonary Medicine, The University of Texas MD Anderson Cancer Center, Houston, TX, United States; ^2^ Tecnologico de Monterrey, Escuela de Medicina y Ciencias de la Salud, Monterrey, Nuevo Leon, Mexico; ^3^ UTHealth Houston Graduate School of Biomedical Sciences, The University of Texas M.D. Anderson Cancer Center, Houston, TX, United States

**Keywords:** COPD, KRAS, toll-like receptor, lung cancer, IKK beta, MyD88

## Abstract

**Introduction:**

Toll-like receptors (TLRs) are an extensive group of proteins involved in host defense processes that express themselves upon the increased production of endogenous damage-associated molecular patterns (DAMPs) and pathogen-associated molecular patterns (PAMPs) due to the constant contact that airway epithelium may have with pathogenic foreign antigens. We have previously shown that COPD-like airway inflammation induced by exposure to an aerosolized lysate of nontypeable *Haemophilus influenzae* (NTHi) promotes tumorigenesis in a K-ras mutant mouse model of lung cancer, CCSP^Cre^/LSL-K-ras^G12D^ (CC-LR) mouse.

**Methods:**

In the present study, we have dissected the role of TLRs in this process by knocking out TLR2, 4, and 9 and analyzing how these deletions affect the promoting effect of COPD-like airway inflammation on K-ras-driven lung adenocarcinoma.

**Results:**

We found that knockout of TLR 2, 4, or 9 results in a lower tumor burden, reduced angiogenesis, and tumor cell proliferation, accompanied by increased tumor cell apoptosis and reprogramming of the tumor microenvironment to one that is antitumorigenic. Additionally, knocking out of downstream signaling pathways, MyD88/NF-κB in the airway epithelial cells further recapitulated this initial finding.

**Discussion:**

Our study expands the current knowledge of the roles that TLR signaling plays in lung cancer, which we hope, can pave the way for more reliable and efficacious prevention and treatment modalities for lung cancer.

## Introduction

Lung cancer continues to lead the way in the context of cancer-related mortality, with over 128,000 deaths expected in the USA alone in 2023 (1). The anatomical and cellular that protect our lungs against exogenous insults such as foreign pathogens and particulates are severely altered in lung cancer. Particularly, when accompanied by inflammatory lung diseases like chronic obstructive pulmonary disease (COPD) (2). Interestingly, current and former smokers with COPD display a 3 to 10-fold increased risk of lung cancer based on their disease severity (3, 4). Importantly, even smokers with COPD who quit smoking will still have persistent inflammation, their lung function will continue to deteriorate, and the risk of lung cancer remains elevated (5–7). Our group has previously shown that COPD-type airway inflammation promotes lung cancer in a K-ras mutant mouse model, this was found to be associated with the activation of NF-ĸB, thus suggesting a role for this pathway in linking COPD to lung cancer (8).

Upstream to the NF-ĸB pathway, there are a group of essential proteins known as toll-like receptors (TLRs) activating the innate immune system due to their high affinity to recognize specific pathogens such as nontypeable *Haemophilus Influenzae* (NTHi) (9). This pathogen in particular is considered the most common colonizer of airways in patients with COPD. TLRs can be of extracellular (TLR-1, TLR-2, TLR-4, TLR-5, TLR-6) or intracellular (s TLR-3, TLR-7, TLR-8, and TLR-9) origin, and participate in different signaling pathways. For example, TLR-3 couples to the adaptor protein TRIF, which ultimately activates TRAF3 and IRF3, and subsequently induces the secretion of IFN-β, a cytokine that is important for the adequate antiviral response. In contrast, other TLRs, like TLR-2, TLR-4, and TLR-9, can bind to a different adapter protein - Myeloid differentiation primary response gene 88 (MyD88). This interaction then causes the recruitment of IL-1 receptor-associated kinases (IRAKs) and TRAF6., which in turn, will phosphorylate and activate the IKK complex leading to the release and translocation of NF-κB to the nucleus and its activation (10, 11) resulting in the synthesis of cytokines such as TNF-α, IL-6, and IL-1β. These are key mediators of the inflammatory response and are known to be elevated in the lung of COPD patients (12). There is however a unique TLR, TLR-4, that activates both MyD88-dependent and TRIF-dependent pathways (13).

TLRs are essential components of the innate immune defense system. They defend our bodies against bacteria, viruses, and other harmful pathogens. However, the role of specific TLRs in the promotion of lung cancer specifically in the context of COPD is not very clear. Previous studies have shown the favorable outcome of TLR knockouts (14), while others have been associated with paradoxical outcomes when manipulated (15). Therefore, we chose to study the role of TLR2, 4, and 9 and their downstream signaling pathways in lung cancer promotion due to their known importance in lung innate epithelial immune response (16–21). We accomplished this using our previously established K-ras mutant lung cancer mouse model in the presence of COPD-like airway inflammation and genetic targeting of the TLR/MyD88/NF-ĸB pathway in this setting. Through these approaches, we have found that TLR2, 4, and 9 through activation of the MyD88/NF-ĸB signaling pathway in the airway epithelium play causal roles in this process. This supports the implementation of modulating TLR signaling for induction of anti-tumor immunity or suppression of pro-tumor inflammation, therefore paving the way for novel immuno-preventive and -therapeutic modalities to ultimately improve patient clinical outcome and survival.

## Methods

### Animal housing and experiments

K-ras mutant mouse model of lung cancer used in this study, CCSP^Cre^/LSL-K-ras^G12D^ (CC-LR), was created by crossing mice harboring LSL-K-ras^G12D^ allele with mice containing Cre recombinase inserted into the Club Cell Secretory Protein (CCSP) locus., This mouse model has K-ras mutant tumor initiation exclusively in lung epithelium as we previously described (8). Then CC-LR mice were crossed with TLR2, TLR4, and TLR9 knockout (KO) mice separately to generate CC-LR/TLR2KO, CC-LR/TLR4KO, and CC-LR/TLR9KO mice respectively. TLR2 (22) and TLR4 KO (23) mice were purchased from Jackson Laboratory, and TLR9 KO was a generous gift from Dr. Shizuo Akira (Osaka University, Japan) (24). These mutant mice globally lack their respective TLR while expressing a mutant K-ras in the lung epithelium. LR/MyD88^Δ/Δ^ and LR/IKKβ^Δ/Δ^ mice, with lung epithelial specific loss of MyD88 and NF-κB activity, were generated by crossing CC-LR with MyD88^fl/fl^ and IKKβ ^fl/fl^ conditional KO mice respectively. IKKβ ^fl/fl^ mouse was kindly provided by Dr. Michael Karin (University of California, San Diego, CA) (25). MyD88^fl/fl^ mouse was purchased from the Jackson Laboratory (26). All the mice used in this study were housed under specific pathogen-free conditions and handled in accordance with the institutional animal care and use committee (IACUC) of the University of Texas MD Anderson Cancer Center. Mice were also monitored daily for evidence of disease or death.

### NTHi lysate preparation and induction of COPD-type airway inflammation

A lysate of NTHi strain 12 (27) was prepared as previously described (28). This was prepared at a protein concentration of 2.5 mg/ml in phosphate-buffered saline (PBS) and stored in 7ml frozen aliquots at −80°C. When delivering the lysate to mice, a thawed aliquot was placed in an AeroMist CA-209 nebulizer (CIS-US, Bedford, MA) driven by 10 liters/minute of room air mixed with 5% CO_2_ for 20 minutes. Mice in all studies were exposed to the lysate once weekly at six weeks of age for a duration of eight weeks, with a final readout at fourteen weeks of age.

### Assessment of lung tumor burden and inflammation

In order to assess lung tumor burden and inflammation, fourteen-week-old mice were anesthetized. Their tracheas were then exposed, cannulated, and sutured into place. If visible, lung surface tumor numbers were counted, then the lungs were perfused with PBS through the right ventricle. For half of the mice in this study, lungs were inflated with 10% buffered formalin (Sigma) for 10 min, then collected and embedded in paraffin blocks for histopathological studies. Formalin fixed paraffin embedded (FFPE) blocks were sectioned at 5-mm thickness, placed on glass slides, dried, deparaffinized, and stained with hematoxylin and eosin. Tumor/lung area percentages were calculated using the respective formula; Tumor/Lung Area =∑i=ni=1TiL × 100%, as described previously (29). For the other half of the mice in this study, bronchoalveolar lavage fluid (BALF) was collected by sequentially instilling and extracting 2ml of PBS through a tracheostomy cannula. Afterward, the lungs were removed and snap-frozen to be stored for RNA and protein analysis. Total WBC counts in BALFs were calculated with the use of a hematocytometer, and differential cell populations (macrophages, neutrophils, and lymphocytes) were determined by cytocentrifugation of BALFs onto slides and Wright–Giemsa (Sigma) staining.

### Immunostaining

Previously sectioned lung tissue samples were immunohistochemically stained as previously described (29) for proliferation marker Ki-67 (1:200; ab16667; Abcam, MA), angiogenesis marker ETS-related gene (ERG) (1:200; ab92513; Abcam) (30), and leukocyte cell marker, CD45 (1:200; ab10558; Abcam). To calculate the percentages of positively stained cells, slides were analyzed using Nuclear v9 in ImageScope 12.4.3 (Leica Biosystems, Nussloch, Germany) and presented as the fraction of positive cells per total tumor cells per 10x field.

### Quantitative RT-PCR analysis

Total RNA from mouse whole lung was extracted using a Zymo Research RNA extraction kit. Reverse transcription PCR was performed using the qScript cDNA SuperMix (Quanta Biosciences, Gaithersburg, MD). We then performed qPCR by using SYBR Green FastMix (Quanta Bioscience) on CFX96 Touch™ Real-Time PCR Detection System (Bio-Rad, Hercules, CA). CD45 was used as our housekeeping gene and data are presented as fold changes of experimental groups versus controls (CC-LR) as indicated in figure legends. Primers used in this study are listed in **Supplementary Table S9**.

### Statistics

Data are presented as mean ± SEM. GraphPad Prism software was used for statistical analysis, and Student’s t-test was used for comparison between every experimental group versus controls (CC-LR). Differences were considered significant for *p < 0.05 and labeled with an asterisk (*), figures with no significant differences were labeled with "ns". Some figures have additional symbols of “#” and “+” to denote different cellular populations as noted in the figure legend.

## Results

### Genetic deletion (knockout) of TLRs suppresses tumor promoting effect of NTHi-induced COPD-like airway inflammation on K-ras driven lung cancer

**Figure 1:**
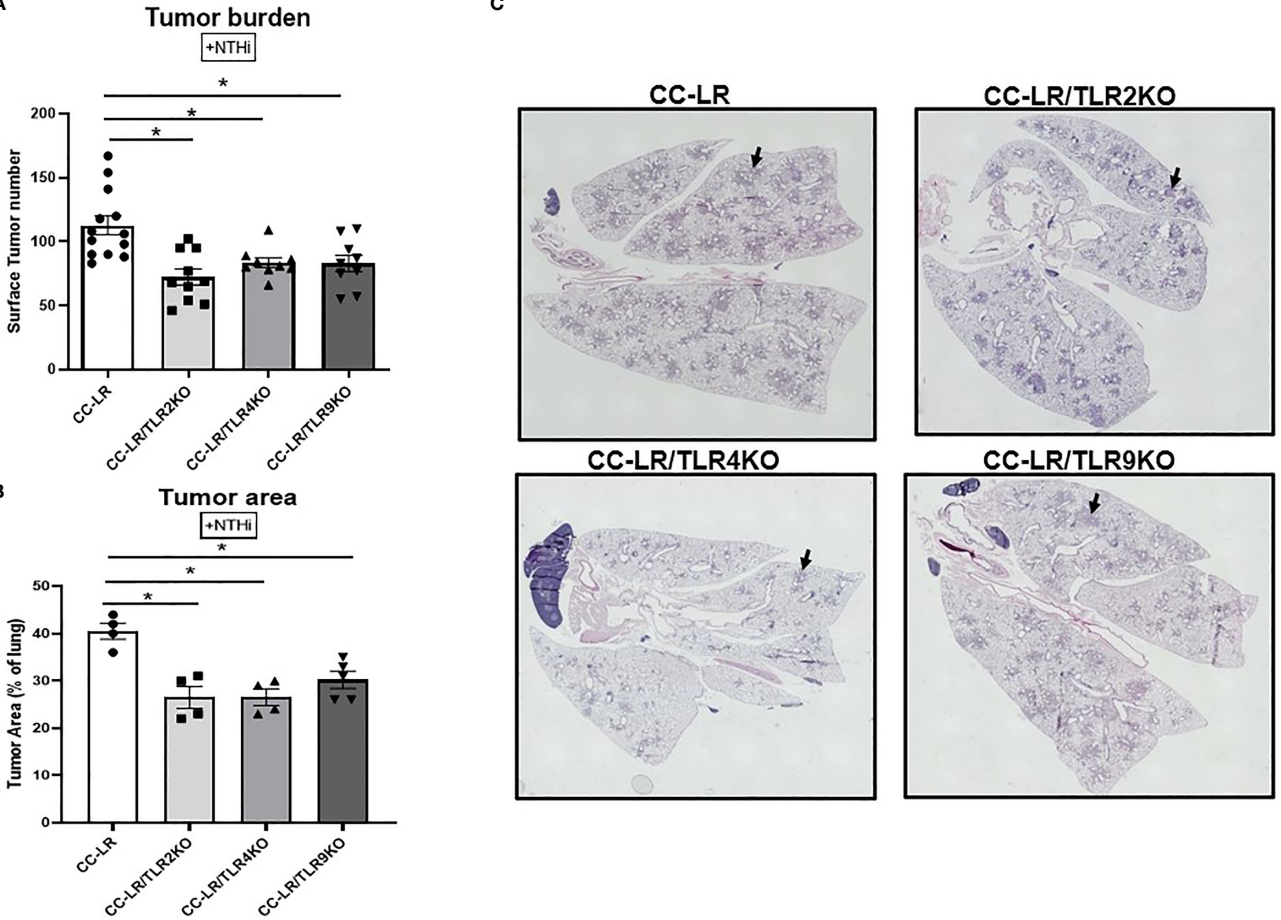
Knockout of TLRs suppresses tumor promoting effect of NTHi-induced COPD-like airway inflammation on K-ras driven lung cancer. Lung surface tumor number (n=9-14) **(A)** and tumor area (n=4-5) **(B)**. Representative photomicrographs of whole slide images of lung hematoxylin and eosin (H&E) stained sections in CC-LR+NTHi, CC-LR/TLR2KO+NTHi, CC-LR/TLR4KO+NTHi, and CC-LR/TLR9KO+NTHi at 14 weeks of age, black arrows depict lung microtumors **(C)**. Data represent mean ± SEM; experimental groups are separately compared to CC-LR cohort, where *p< 0.05 by unpaired t-test.

The exact role of TLRs in lung cancer promotion is not well established, therefore we sought to study the role of TLRs by crossing CC-LR mice with TLR2, TLR4, and TLR9 null mice separately to generate K-ras mutant mice lacking TLR2, TLR4, and TLR9, respectively (CC-LR/TLR2KO, CC-LR/TLR4KO, CC-LR/TLR9KO). These cohorts were then subjected to COPD-inducing conditions from the age of 6 weeks for 8 weeks as described above and previously (8) and their tumor burdens were compared with age and sex matched control CC-LR mice at the age of 14 weeks. The lack of TLRs in these three distinct mutant mouse models significantly inhibited lung cancer promotion by COPD-like airway inflammation in CC-LR mice. Lung surface tumor numbers decreased by 1.5-fold (72 vs. 112) in CC-LR/TLR2KO, 1.3-fold (84 vs. 112) in CC-LR/TLR4KO, and 1.3-fold (83 vs. 112) in CC-LR/TLR9KO mice (**Figure 1A**). Tumor area analysis of H&E-stained slides also showed a 1.5-fold (27% vs. 41%) reduction in CC-LR/TLR2KO and CC-LR/TLR4KO, while CC-LR/TLR9KO showed a 1.4-fold reduction (29% vs. 41%) in the lung area occupied by the tumors (**Figures 1B, C**). We also studied the role of TLRs in lung cancer promotion in mice lacking COPD-inducing conditions (Naïve). The naïve cohorts of CC-LR/TLR2, 4, and 9 mice also showed 1.7-fold (35 vs 60), 1.5-fold (40 vs. 60), and 2-fold (30 vs. 60) reductions in tumor numbers respectively compared to age and sex match naïve CC-LR mice (**Supplementary Figure S1**). In these naïve cohorts, we also observed a significant 1.5-fold reduction (17% vs. 28%) across all three mouse models in the lung area occupied by the tumors (**Supplementary Figure S1**). These findings collectively confirm the protumorigenic effects of these TLRs in K-ras mutant lung cancer development and promotion.

### Lung cancer suppression due to knockout of TLRs is associated with decreased tumor cell proliferation and angiogenesis

**Figure 2:**
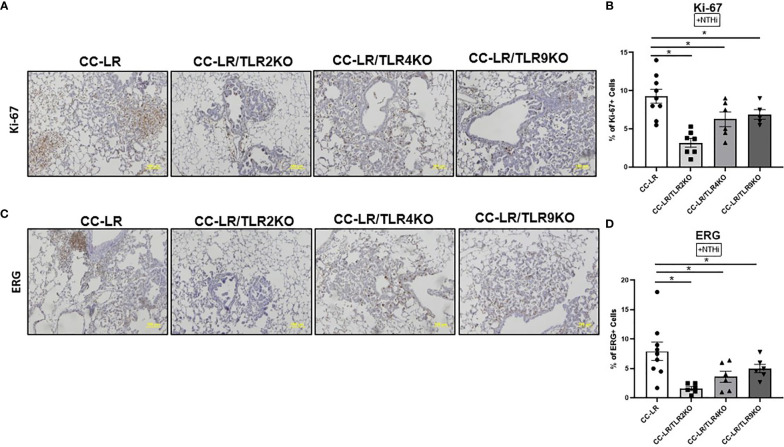
Lung cancer suppression due to TLR knockout is associated with decreased proliferation and angiogenesis. Representative photomicrographs of lung immunohistochemistry that are positive for Ki-67 **(A)**, and ERG **(C)** (20x magnification, scale bar=100um). Accompanied by quantitative analysis (right panels) represented as a percentage of positive KI-67 (n=4-5) **(B)** and ERG cells (n=6-9) **(D)**. Data represent mean ± SEM; experimental groups are separately compared to CC-LR cohort, where *p< 0.05 by unpaired t-test.

We aimed to further study the mechanism of tumor reduction in mice with a lack of TLRs and COPD-like airway inflammation. Lung tissues were examined *via* immunohistochemistry for proliferation, and angiogenesis markers, Ki-67, and ERG respectively. Ki-67 staining of the lung from CC-LR/TLR4KO and CC-LR/TLR9KO mutant mouse models showed a reduction in tumor cell proliferation by ~15%, while CC-LR/TLR2KO had a greater reduction of 50% in Ki-67 positive cells (**Figures 2A, B**). We also found that TLR knockout mice had reduced angiogenesis. CC-LR/TLR4KO and CC-LR/TLR9KO mutants showed a 30% reduction and CC-LR/TLR2KO a 75% reduction in ERG positive cells (**Figures 2C, D**). Our naïve cohort of mice displayed a low baseline of Ki-67/ERG positivity and showed significant reductions for both markers. TLR2, 4, and 9 displayed an 80% reduction in Ki-67 positivity, while TLR2 and 4 had a 65% reduction and TLR9 had a 35% reduction in ERG positivity (**Supplementary Figure S2**). Indeed, these data hint at the promoting effects that TLRs may have on tumor cell proliferation and angiogenesis.

### Knockout of TLRs reprograms COPD-associated protumorigenic inflammatory phenotype into an antitumorigenic phenotype

**Figure 3:**
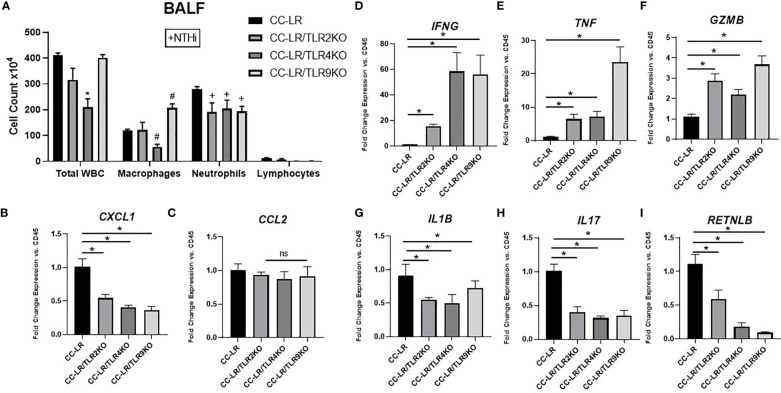
Knockout of TLRs reprograms COPD-associated protumorigenic inflammatory phenotype into an antitumorigenic phenotype. Total inflammatory cell and lineage-specific leukocyte numbers from BALF (n=6-8) **(A)**. Relative mRNA expression of *CXCL1*, *CCL2*, *IFNG*, *TNF*, *GZMB*, *IL1B*, *IL17*, and *RETNLB* mRNA in whole lungs, normalized by *CD45* expression (n=4-6) **(B-I)**. Data represent mean ± SEM; experimental groups are separately compared to CC-LR cohort, where "*", "#", "+" denote p< 0.05 by unpaired t-test of Total WBC, Macrophages, and Neutrophils respectively.

Here, we sought to elucidate the intricate mechanisms of tumor suppression due to TLR knockouts in the setting of COPD-like airway inflammation, specifically regarding changes in the tumor microenvironment (TME) and tumor-infiltrating immune subsets. We accomplished this by analyzing the BALFs of TLR2, 4, and 9 KO/K-ras mutant mouse models in the presence of NTHi-induced COPD-like airway inflammation. We found that all three of these TLR KO mice had a significant reduction in lung neutrophilic infiltrates (**Figure 3A**). There were no significant changes in lymphocytes, although CC-LR/TLR4KO showed a significant decrease in macrophages, and CC-LR/TLR9KO showed a significant increase (**Figure 3A**). Additionally, mRNA expression analysis of whole lungs from these KO mice revealed significant reductions of neutrophil chemoattractant *CXCL1*, with a 2-fold decrease in TLR2 KO and a 2.5-fold decrease in TLR4 or 9 KO mice (**Figure 3B**). No significant changes in macrophage chemoattractant *CCL2* were found (**Figure 3C**). These results were accompanied by significantly increased levels of *IFNG*, specifically a 15-fold increase in TLR2 KO and a 50-fold increase in TLR4 or 9 KO mice (**Figure 3D**). *TNF* also displayed significant increases of 7-fold in TLR2 or 4 KO and 20-fold in TLR9 KO mice (**Figure 3E**), and *Gzmb* showed a 2.5-fold increase in TLR2, a 2-fold increase in TLR4 and a 3-fold increase in TLR9KO mice (**Figure 3F**). *IL1*β revealed a 1.5-fold reduction in TLR2/4, and a 1.4-fold reduction in TLR9 KO mice (**Figure 3G**), while *IL17* showed a homogenous 2.5-fold reduction across all TLR KO mice (**Figure 3H**). Lastly, protumorigenic and immunosuppressive macrophage polarization marker *RETNLB* (Fizz1) showed a significant 2-fold reduction in TLR2 KO, a 5-fold reduction in TLR4 KO, and a 10-fold reduction in TLR9 KO mice (**Figure 3I**). To further explore the unique immune cell changes across the different TLR KO mice in the context of NTHi-mediated inflammation, we studied the mRNA expression levels of different immune cell markers including total leukocytes (*CD45*), T-cells (*CD3*), neutrophils/granulocytes (*Ly6G*), cytotoxic T-cells (*CD8*) and monocytes (*CD68*). We also performed immunohistochemistry for *CD45*, which represents all immune cell types in our study. Results from mRNA expression of *CD45, CD3, CD8, and CD68* showed no significant changes across TLR mutant mice, the only significant change was *Ly6G* which displayed a significant decrease in TLR2KO (**Supplementary Figure S3A-E**). Additionally, our immunohistochemistry studies showed a significant reduction in CD45 staining across all TLR KO mouse models, further supporting a decrease in pro-tumor inflammatory TME of these mice (**Supplementary Figure S3F, G**).

In comparison, our naïve cohort of mice displayed a distinct phenotype. There were no significant changes to lymphocytes or neutrophils, although all three mouse models showed a significant increase in macrophages. *CXCL1* presented with a 1.6-fold reduction across all three mouse groups. No significant changes were seen for *CCL2* or *Gzmb*. *TNF* showed a significant 4-fold increase for TLR2, and 4, and a 5-fold increase for TLR9. *IFNG* had a 3-fold increase for TLR2, a 9-fold increase for TLR4, and a 5-fold increase for TLR9. This was accompanied by significant reductions in *IL1β*, 2.5-fold reduction for TLR2, 1.6-fold reduction for TLR4, and 1.2-fold reduction for TLR9. *IL17* results showed that all three groups shared a significant 1.6-fold reduction. Lastly, *RETNLB* displayed that TLR2 had a 1.6-fold reduction and TLR4 and 9 had a 2-fold reduction (**Supplementary Figure S4**). In summary, these findings further support a shift to an antitumorigenic TME phenotype in response to the lack of TLRs.

### Targeting downstream signaling pathways to TLRs recapitulates the effect of TLR-knockout on the promotion of K-ras driven lung cancer by COPD-like airway inflammation

**Figure 4:**
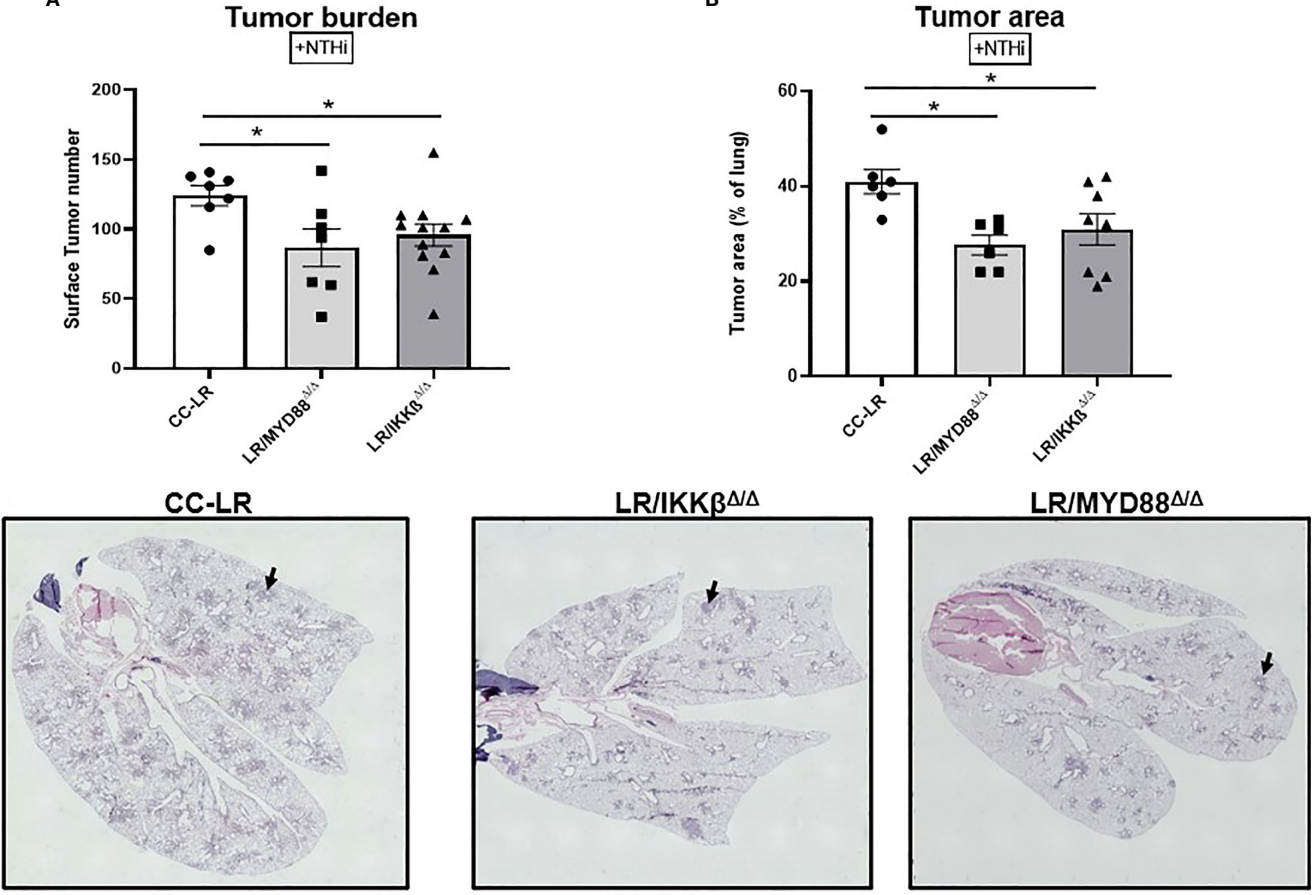
Targeting downstream signaling pathways to TLRs recapitulates the effect of TLR-knockout on the promotion of K-ras driven lung cancer by COPD-like airway inflammation. Lung surface tumor number (n=7-12) **(A)** and tumor area (n=6-7) **(B)**. Representative photomicrographs of whole slide images of lung hematoxylin and eosin (H&E) stained sections in CC-LR, LR/MYD88^Δ/Δ^, and LR/IKKβ^Δ/Δ^ mice at 14 weeks of age, black arrows depict lung microtumors **(C)**. Data represent mean ± SEM; experimental groups are separately compared to CC-LR cohort where *p< 0.05 by unpaired t-test.

To further dissect the cell type specific mechanism of tumor promotion by TLR activation in a COPD setting, we studied the signaling checkpoints shared among TLRs, specifically MyD88/NF-κB signaling. To do this, we developed CC-LR mice with airway specific deletion of MyD88 (LR/MyD88^Δ/Δ^), and IKKβ (LR/IKKβ^Δ/Δ^) separately, then exposed them to COPD-inducing conditions like TLR KO models. Interestingly, we found a robust reduction in tumor burden similar to the TLR KO models. LR/MyD88^Δ/Δ^ had a 1.4-fold reduction (87 vs. 124) and LR/IKKβ^Δ/Δ^ had a 1.3-fold (96 vs. 124) reduction in lung surface tumor numbers compared to age and sex match CC-LR control mice in the presence of COPD-like airway inflammation (**Figure 4A**). Tumor area analysis revealed a significant 1.5-fold (27% vs. 41%) and 1.3-fold (31% vs. 41%) reduction in LR/MyD88^Δ/Δ^ and LR/IKKβ^Δ/Δ^ mice, respectively (**Figures 4B, C**). Our naïve cohort of mice also presented with a significant 1.8-fold reduction (30 vs. 55) in tumor burden for both LR/MyD88^Δ/Δ^ and LR/IKKβ^Δ/Δ^ mice (**Supplementary Figure S5**). These findings in naïve mice were accompanied by a 1.5-fold reduction (18% vs 28%) and an 85% reduction in both LR/MyD88^Δ/Δ^ and LR/IKKβ^Δ/Δ^ (**Supplementary Figure S5**). These findings support our initial studies with TLR KO mice and suggest downstream signaling, specifically the MyD88/NF-κB pathway is essential in COPD-driven K-ras mutant lung cancer promotion.

### Lung cancer suppression due to epithelial specific knockout of MyD88/NF-κB signaling is associated with decreased tumor cell proliferation and angiogenesis

**Figure 5:**
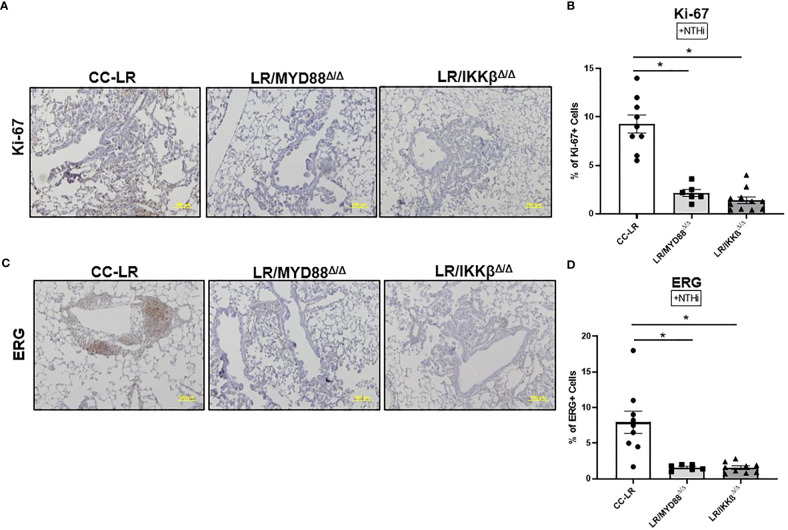
Targeting downstream signaling pathways to TLRs recapitulates the effect of TLR-knockout on the promotion of K-ras driven lung cancer by COPD-like airway inflammation. Representative photomicrographs of lung immunohistochemistry that are positive for Ki-67 **(A)**, and ERG **(C)** (20x magnification, scale bar=100um). Accompanied by quantitative analysis (right panels) represented as a percentage of positive Ki-67 (n=7-10) **(B)** and ERG cells (n=5-6) **(D)**. Data represent mean ± SEM; experimental groups are separately compared to CC-LR cohort, where *p< 0.05 by unpaired t-test.

Similar to TLR KO models, we sought to study the mechanism of epithelial specific knockouts of MyD88/NF-κB in K-ras mutant lung tumor promotion. Thus, we analyzed lung tissue for markers of cell proliferation and angiogenesis. LR/MyD88^Δ/Δ^ and LR/IKKβ^Δ/Δ^ mice showed a robust decrease in Ki-67 positive cells of about 80% (**Figures 5A, B**) and ERG positive cells of about 85% (**Figures 5C, D**). Upon analyzing our naïve cohort of mice, we found Ki-67 positive cells displayed a significant 55% reduction for LR/MyD88^Δ/Δ^ and an 85% reduction for LR/IKKβ^Δ/Δ^ mice. On the other hand, ERG had approximately a 30% reduction in LR/MyD88^Δ/Δ^ and an 80% reduction in LR/IKKβ^Δ/Δ^ mice (**Supplementary Figure S6**). These results indicate that TLR downstream signaling pathways may play a role in regulating tumor cell proliferation and angiogenesis.

### Knockout of epithelial specific MyD88/NF-κB signaling reprograms COPD-associated protumorigenic inflammatory phenotype into an antitumorigenic phenotype

**Figure 6:**
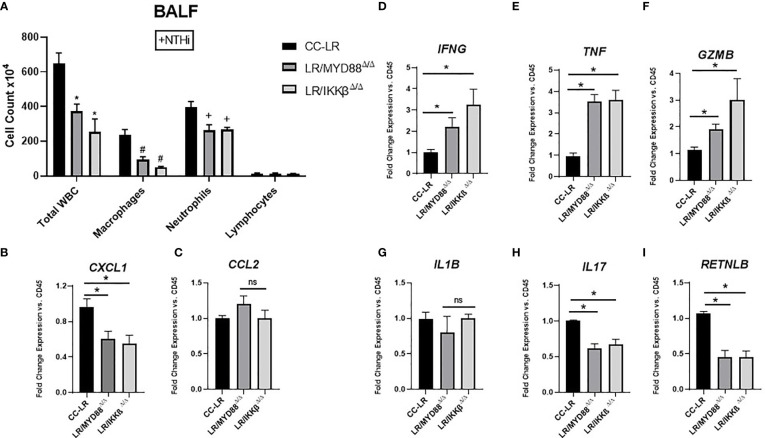
Knockout of epithelial specific MyD88/NF-ĸB signaling reprograms COPD-associated protumorigenic inflammatory phenotype into an antitumorigenic phenotype. Total inflammatory cell and lineage-specific leukocyte numbers from BALF (n=5-7) **(A)**. Relative mRNA expression of *CXCL1*, *CCL2*, *IFNG*, *TNF*, *GZMB*, *IL1B*, *IL17*, and *RETNLB* in whole lungs, normalized by *CD45* expression (n=4-5) **(B-I)**. Data represent mean ± SEM; experimental groups are separately compared to CC-LR cohort, where "*", "#", "+" denote p< 0.05 by unpaired t-test of Total WBC, Macrophages, and Neutrophils respectively.

To gain insight into the immune cell phenotype of K-ras mutant MyD88 or IKKβ conditional knockout models, we studied the BALF inflammatory cells along with protumorigenic and anti-tumorigenic cytokine milieu. We found evidence of a similar reprogramming in the immune cell phenotype as TLR KOs, characterized by a reduction in total WBC counts driven mainly by a reduction in neutrophils followed by a significant reduction in the macrophage population (**Figure 6A**). Interestingly, neutrophil chemoattractant *CXCL1* showed a 1.6-fold decrease in both LR/MyD88^Δ/Δ^ and LR/IKKβ^Δ/Δ^ mice (**Figure 6B**), however, macrophage chemoattractant *CCL2* did not show significant changes (**Figure 6C**). IFNG displayed a significant 2-fold increase in LR/MyD88^Δ/Δ^ and a 3-fold increase in LR/IKKβ^Δ/Δ^ mice (**Figure 6D**). There was a 3.5-fold increase in *TNF* levels in both LR/MyD88^Δ/Δ^ and LR/IKKβ^Δ/Δ^ mice (**Figure 6E**), and *Gzmb* showed a 1.9-fold increase in LR/MyD88^Δ/Δ^ and 2.7-fold increase in LR/IKKβ^Δ/Δ^ mice (**Figure 6F**). *IL-1β* was unaffected (**Figure 6G**), while, *IL17* revealed a 1.4-fold decrease in both KO mice (**Figure 6H**). Consistent with our findings in TLR KO mice, we also found a significant 2-fold decrease in protumorigenic/immunosuppressive marker *RETNLB* (Fizz1) in both LR/MyD88^Δ/Δ^ and LR/IKKβ^Δ/Δ^ mice (**Figure 6I**). In contrast to TLR KOs, *CD45* mRNA expression in LR/MyD88^Δ/Δ^ and LR/IKKβ^Δ/Δ^ mice showed a significant decrease, which is consistent with the relatively lower number of leukocytes present in these mice compared to TLR KOs (**Supplementary Figure S7A**). T-cell marker *CD3* and more specifically cytotoxic T-cell marker *CD8* both showed a significant increase in LR/MyD88^Δ/Δ^ and LR/IKKβ^Δ/Δ^ (**Supplementary Figure S7D, E**), possibly due to the fact that MyD88 and IKKβ are downstream to multiple TLRs and blocking them leads to a signaling response akin to the promotion of T-cell cytotoxicity, which is nicely supported by the observed mRNA expression levels of *Gzmb* in these cohorts. *CD68* expression showed no significant changes, while *Ly6G* was significantly decreased in LR/IKKβ^Δ/Δ^ mutant mice (**Supplementary Figures S7B, C**). Interestingly, IHC results were similar to TLR KO mice, supporting the previous notion of a less inflamed TME as we found a significant decrease of CD45-positive staining across all mutant mice models (**Supplementary Figures S7F, G**).

In the naïve cohorts of LR/MyD88^Δ/Δ^ and LR/IKKβ^Δ/Δ^, like our naïve TLR KO cohort we found no significant changes in either neutrophils or lymphocytes. However, contrary to our naïve TLR KO cohort, targeting these downstream signaling pathways led to significant reductions in macrophages. Interestingly, *CXCL1*, *CCL2*, and *Gzmb* showed no significant changes. *TNF* levels in both LR/MyD88^Δ/Δ^ and LR/IKKβ^Δ/Δ^ naïve mice had a significant 3.5-fold increase. *IFNG* displayed a 2.2-fold increase for LR/MyD88^Δ/Δ^ and a 3-fold increase for LR/IKKβ^Δ/Δ^. Expression of *IL-1β* displayed a 1.7-fold and 1.2-fold reduction in naïve LR/MyD88^Δ/Δ^ and LR/IKKβ^Δ/Δ^ respectively. Lastly, *IL17* had a significant 1.4-fold reduction for both mouse models of downstream TLR signaling pathways and *RETNLB* had a 2-fold reduction as well (**Supplementary Figure S8**). Ultimately, these findings in conjunction with our initial TLR KO models validate an antitumorigenic phenotype that the knockout of TLRs or epithelial specific knockout of MyD88/NF-κB could induce.

## Discussion

TLRs can recognize various antigens, unleashing a signaling cascade through MyD88, IKKβ interaction, and ultimately activating the NF-κB pathway, a prominent promoter of inflammation and tumorigenesis (31, 32).

The study presented here shows that complete knockout of either TLR 2, 4, and 9 suppresses the tumor promoting effect of COPD-like lung inflammatory microenvironment, with CC-LR/TLR2KO seeming to manifest the strongest phenotype owing to the highest reduction in tumor burden and accompanied changes in TME. Additionally, our findings support that the epithelial cell specific knockout of downstream signaling pathways to TLRs, MyD88, and IKKβ, also suppresses the tumor promoting effect of COPD-like lung inflammatory microenvironment.

Accordingly, we conclude that the observed antitumorigenic effect of targeting TLRs or epithelial specific MyD88/IKKβ is mainly due to the inhibition of the NF-κB pathway, and subsequent changes in the gradient of essential protumor versus anti-tumor cytokines as we and others have previously shown (8, 28, 33–35). Some of these cytokines can also influence cell proliferation, vascular permeability, and macrophage migration (35–37). Interestingly, these cytokines remain elevated for a longer period during TLR2 activation than in TLR4 activation (38). TLR4 function is similar to TLR2 because of the common signaling checkpoint (MyD88) they share, and activating NF-κB pathway, which could lead to tumor cell proliferation, apoptosis inhibition, and metastasis (39). TLR9 is unique due to being located intracellularly, where it detects viral/bacterial nucleic acid (particularly CpG DNA) in late endosomes-lysosomes (40, 41). Previous studies have proven TLR9’s tumorigenic role *in vivo* in NSCLC through the upregulation of VEGF and iNOS, thus promoting an angiogenic phenotype (42). Among the TLRs and their downstream (MyD88/IKKβ) we studied here, all had pro-tumor inflammatory functions, because their knockout led to reduced tumor burden, reduced angiogenesis, and reduced cell proliferation. Importantly, we observed a decrease in COPD driven lung neutrophilic influx which we and others have shown to have an essential role in the promotion of K-ras mutant lung cancer (8, 43–45).

Macrophages are an important component of the inflammatory TME. Previous studies have shown the roles that TLR3/TLR4 have in promoting an M1 antitumor macrophage phenotype (46, 47). In this study, we found a predominant protumorigenic myeloid cell phenotype (M2), and have shown reprogramming into one that is antitumorigenic in response to lack of TLR/MyD88/NF-kB pathway. Specifically, we found that TLR 2, 4, and 9 knockouts, as well as epithelial specific knockout of MyD88/NF-κB pathway, have a significant effect on reducing expressions of protumorigenic immunosuppressive factors such as RETNLB, and induction of anti-tumorigenic cytotoxic factors like IFNγ along with increased apoptosis (Gzmb) possibly *via* TNF-mediated cleaved caspase 3/8 activity. These results are further supported by previous studies dissecting the mechanism of TLR-mediated apoptosis through the RIP1 signaling pathway, where authors have shown that TLRs can activate the RIP/FADD/caspase-8-axis through their common adaptor protein TRIF to ultimately promote cell death (48–50).

Our study is set in a TME that is heavily inflamed due to neutrophilic influx *via* robust COPD-mediated TLR activation. The primary insult caused by pathogens like NTHi, which is the most prevalent pathogen that exists in the lung of patients with COPD (51, 52), and other inflammatory stimuli like cigarette smoke gives rise to the most common phenotypic inflammatory characteristic of COPD, neutrophilic influx. This is known to have a positive correlation with disease severity as well as lung cancer progression (43, 53, 54). Interestingly, an important hallmark of our study is the attenuation of COPD associated neutrophilic influx in the absence of TLRs or their epithelial specific downstream signaling pathways which drives the favorable outcomes present in this context. This could be mainly described by the observed changes in the expression of neutrophil chemoattractants such as CXCL1 that are known to be modulated by the NF-ĸB pathway. It is important to consider that this phenomenon could also be partly due to the significant decrease in IL-17 which we have found in our study. IL-17 is a cytokine that has been shown by us to be a key player in promotion of K-ras mutant lung tumorigenesis by NTHi-induced COPD type lung inflammation (55), as well as by others where it was specifically found to be dependent on TLR2/4 in the context of NTHi-mediated inflammation (56).

It is also worth noting our favorable findings related to naïve tumorigenesis (mice without exposure to NTHi) present in our study, which is probably due to a positive feedback loop in tumor cells. As discussed earlier, we and others have previously reported that K-ras mutant tumors have an intrinsic inflammatory phenotype that is associated with the activation of the NF-kB pathway and production of a group of inflammatory pro-tumor cytokines (8, 51, 57). Consequently, the increased expression of these cytokines by tumor cells could signal to immune cells or have an intrinsic effect on tumor cells, therefore explaining the observed effect of TLR/IKKβ/MyD88 knockout on naïve tumorigenesis.

Taken together, these data suggest a link between TLR-mediated inflammation in COPD and lung cancer progression, introducing TLR/MyD88/NF-kB pathway as a key player in linking COPD to lung cancer. Interestingly, the use of TLR antagonists in lung cancer is lacking. Multiple studies demonstrate that TLR agonists improve cancer outcomes (15, 58–60). However, our study suggests that the observed antitumor response after TLR/MyD88/NF-κB pathway inhibition is mediated by a delicate balance between immune activation and immune suppression in the tumor microenvironment (TME), It provides a baseline for the development of alternative intervention modalities., and paves the way for the introduction of agents inhibiting innate immune signals such as TLRs for prevention and treatment of K-ras mutant lung cancer in high-risk individuals (Smokers with and without COPD).

## Data availability statement

The original contributions presented in the study are included in the article/**Supplementary Materials**, further inquiries can be directed to the corresponding author.

## Ethics statement

The animal study was reviewed and approved by University of Texas MD Anderson Cancer Center Institutional Animal Care and Use Committee.

## Author contributions

SM conceived, designed, supervised and conceptualized the study; WV, MR-C, NK, SC-P, MG, AK, MC, NT-G, MU, SA, DDB, SD, and MDLG performed research; WV and SM analyzed data; SE provided reagents, WV and SM wrote the paper. All authors contributed to the article and approved the submitted version.
