# Supplementary material for: Toll-like receptors 2, 4, and 9 modulate promoting effect of COPD-like airway inflammation on K-ras-driven lung cancer through activation of the MyD88/NF-ĸB pathway in the airway epithelium
Source: Front Immunol. 2023 May 22;14:1118721. doi: 10.3389/fimmu.2023.1118721 (PMC10240392; doi:10.3389/fimmu.2023.1118721)
Supplement: Supplementary file 1 [file DataSheet_1.docx]

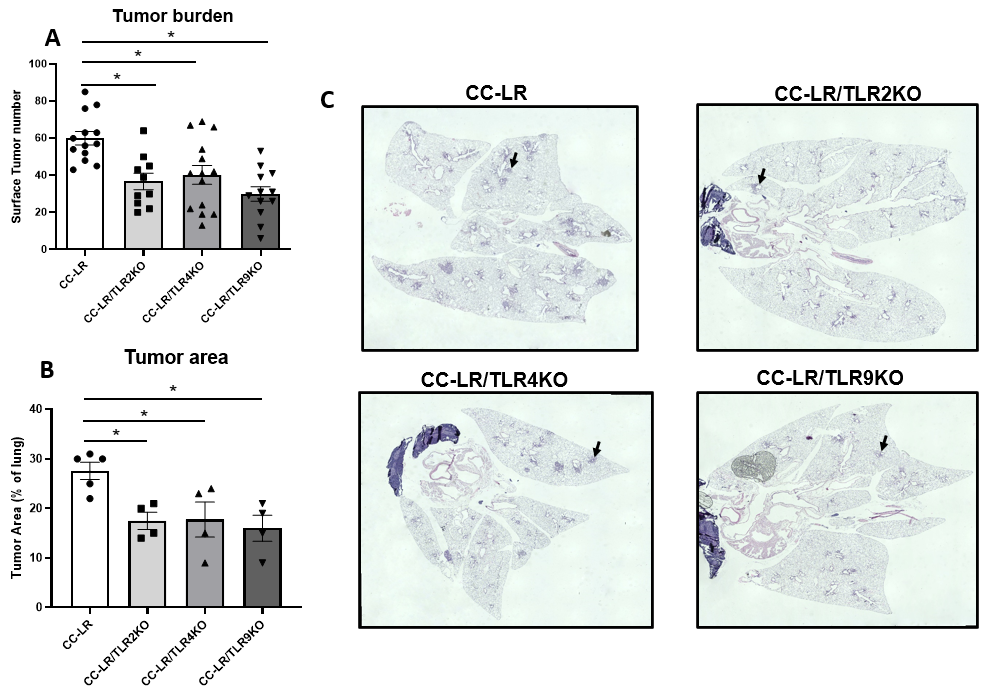


**Supplementary Figure S1. Tumor burden in naïve TLR KO models.** Lung surface tumor count (n=12-14) (A). Tumor/lung area percentage (4-5) (B). Representative photomicrographs of the whole slide images of H&E-stained sections, and black arrows depict lung microtumors (C). Data represent mean ± SEM; experimental groups are separately compared to the CC-LR cohort alone, where *p< 0.05 by unpaired t-test.


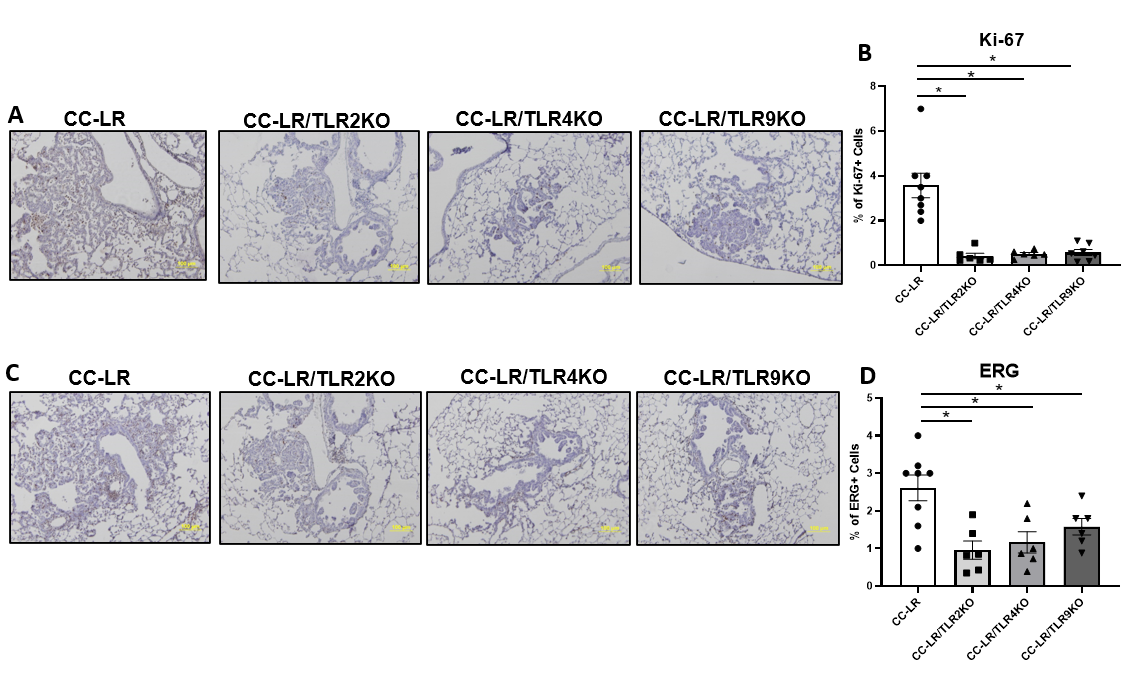


**Supplementary Figure S2**. **Ki-67 and ERG staining in naïve TLR KO models.** Representative photomicrographs of Ki-67-stained sections (n=6-8) (A), and its quantification (B). Representative photomicrographs of ERG-stained sections (n=6-8) (C) and its quantification (D). Data represent mean ± SEM; experimental groups are separately compared to the CC-LR cohort alone, where *p< 0.05 by unpaired t-test.


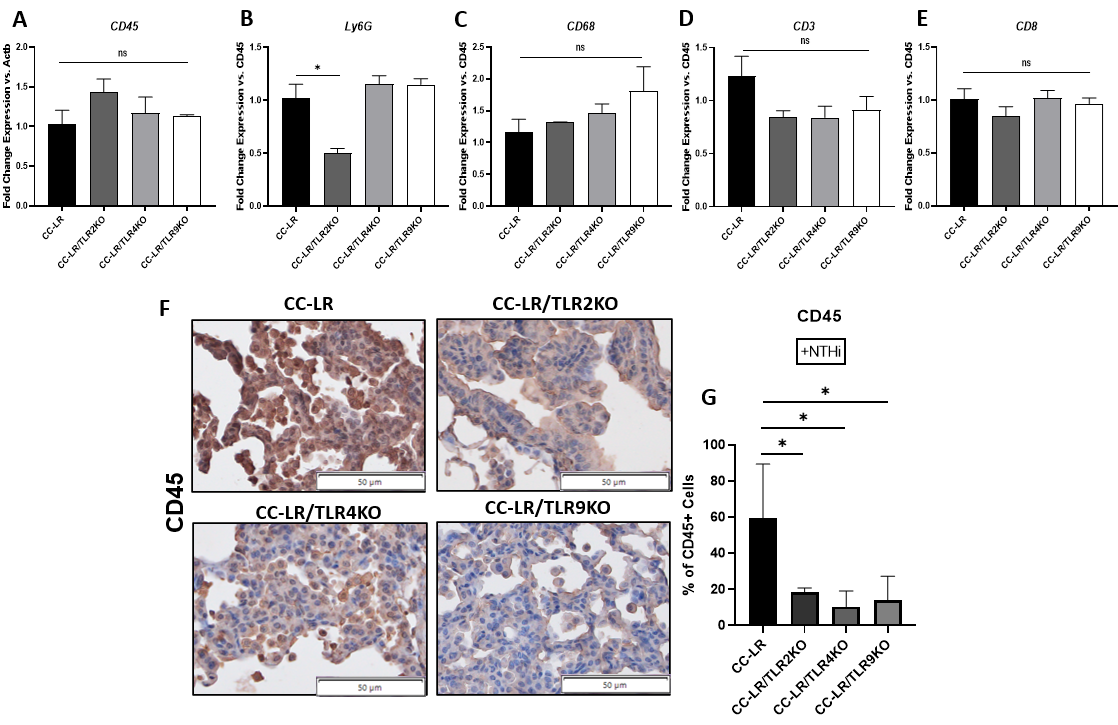


**Supplementary Figure S3. TLR KO immune cell phenotyping.** Relative CD45 mRNA expression in whole lungs normalized to Actb (n=3) (A) Ly6G, CD68, CD3, and CD8 mRNA expression normalized to CD45 expression (n=3) (B-E). Representative photomicrographs of CD45-stained sections (n=3) (F), and its quantification (G). Data represent mean ± SEM; experimental groups are separately compared to the CC-LR cohort alone, where *p< 0.05 by unpaired t-test.


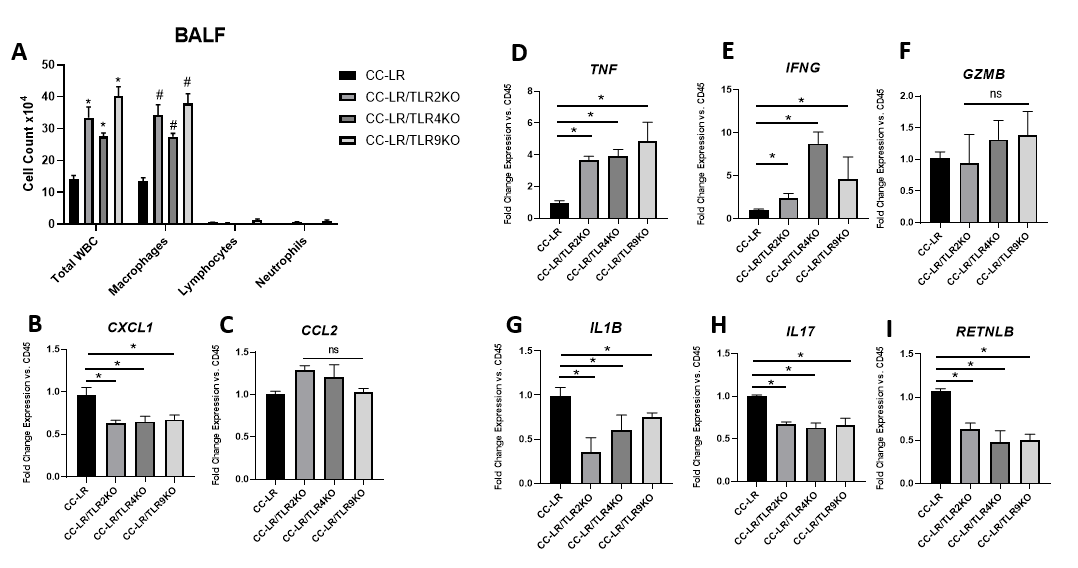


**Supplementary Figure S4.** **Bronchoalveolar lavage fluid** **(BALF) infiltrates quantification and characterization in naïve TLR KO models.** Total inflammatory cell and lineage-specific leukocyte numbers in BALFs (n=8-10) (A). Relative mRNA expression of CXCL1, CCL2, IFNG, TNF, GZMB, IL1B, IL17, and RETNLB mRNA in whole lungs, normalized by CD45 expression (n=4-5) (B-I). Data represent mean ± SEM; experimental groups are separately compared to the CC-LR cohort alone, where *p< 0.05 by unpaired t-test.


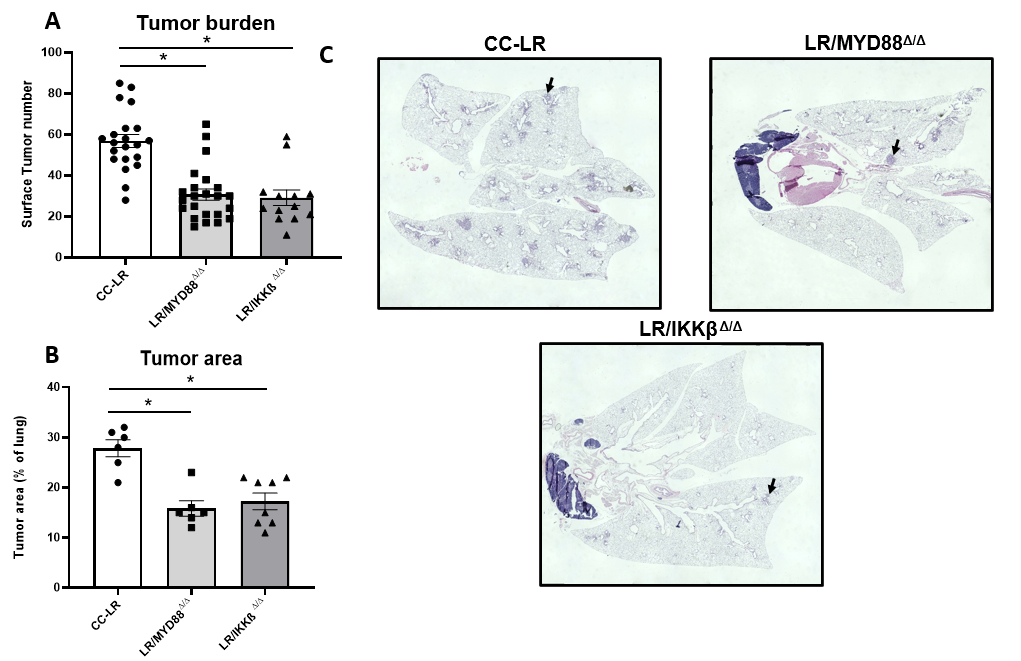


**Supplementary Figure S5. Tumor burden in naïve LR/MYD88^Δ/Δ^ and LR/IKKβ^Δ/Δ^ models.** Lung surface tumor count (n=12-21) (A). Tumor/lung area percentage (n=6-8) (B). Representative photomicrographs of the whole slide images of H&E-stained sections, and black arrows depict lung microtumors (C). Data represent mean ± SEM; experimental groups are separately compared to the CC-LR cohort alone, where *p< 0.05 by unpaired t-test.


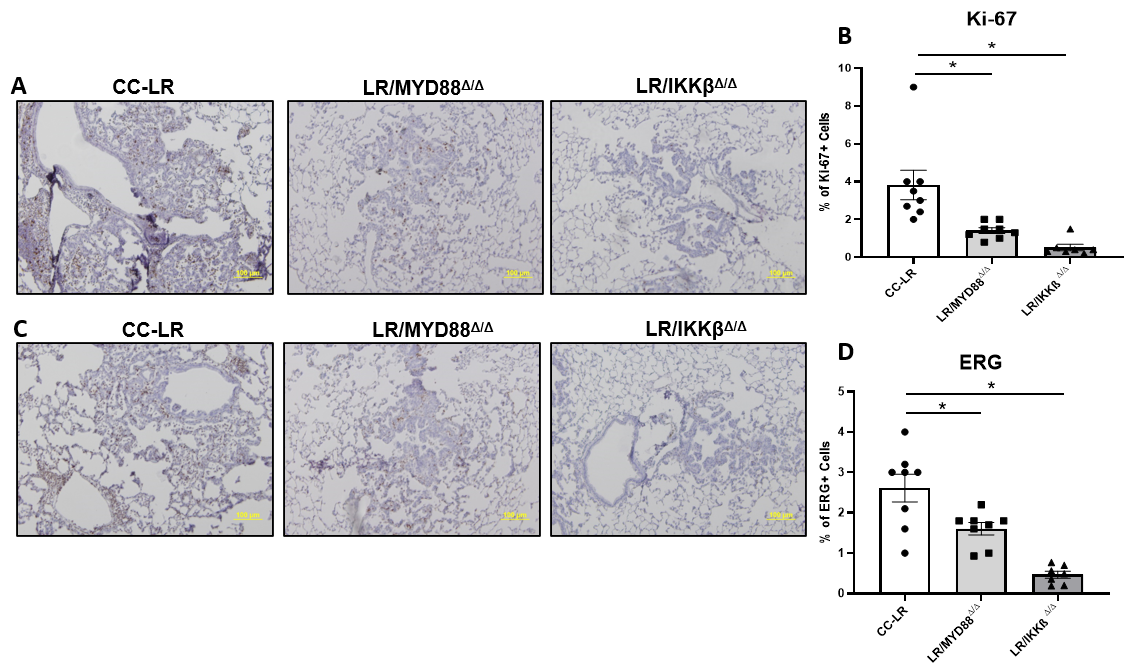


**Supplementary Figure S6. Ki-67 and ERG staining in naïve LR/MYD88^Δ/Δ^ and LR/IKKβ^Δ/Δ^ models.** Representative photomicrographs of Ki-67-stained sections (n=7-8) (A), and its quantification (B). Representative photomicrographs of ERG-stained sections (n=7-8) (C) and its quantification (D). Data represent mean ± SEM; experimental groups are separately compared to the CC-LR cohort alone, where *p< 0.05 by unpaired t-test.


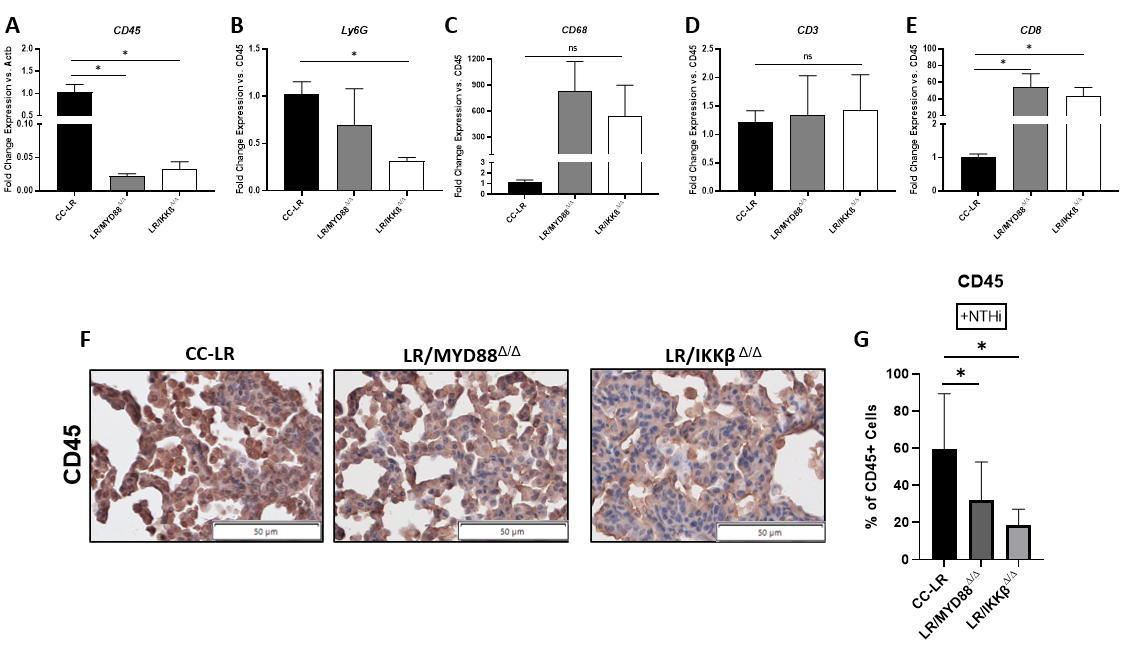


**Supplementary Figure S7. LR/MYD88^Δ/Δ^ and LR/IKKβ^Δ/Δ^ immune cell phenotyping.** Relative CD45 mRNA expression in whole lungs normalized to Actb (n=3) (A). Ly6G, CD68, CD3, and CD8 mRNA expression normalized to CD45 expression (n=3) (B-E). Representative photomicrographs of CD45-stained sections (n=3) (F), and its quantification (G). Data represent mean ± SEM; experimental groups are separately compared to the CC-LR cohort alone, where *p< 0.05 by unpaired t-test.


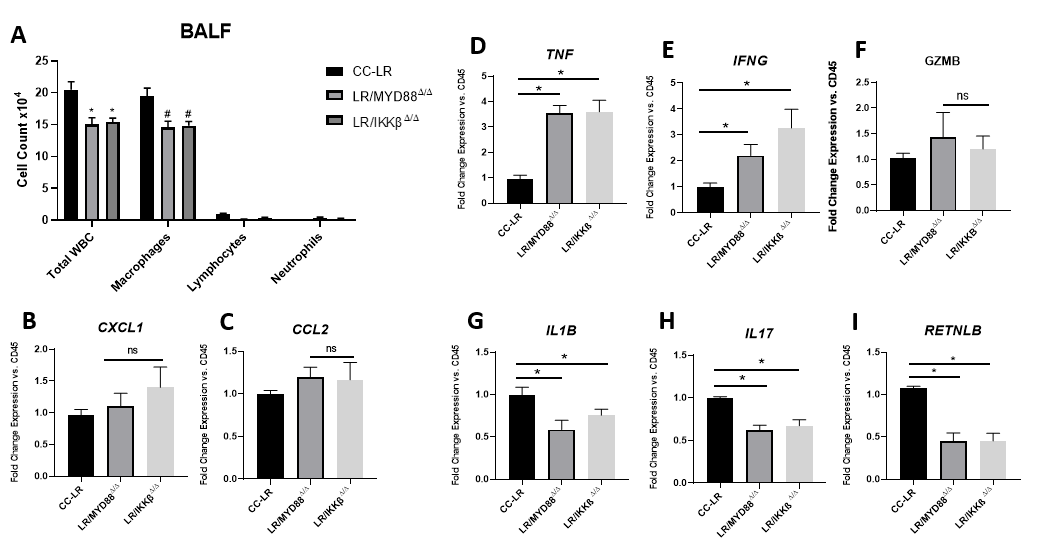


**Supplementary Figure S8. Bronchoalveolar lavage fluid** **(BALF) infiltrates quantification and characterization in naïve LR/MYD88^Δ/Δ^ and LR/IKKβ^Δ/Δ^ models**. Total inflammatory cell and lineage-specific leukocyte numbers in BALFs (n=7-9) (A). Relative mRNA expression of CXCL1, CCL2, IFNG, TNF, GZMB, IL1B, IL17, and RETNLB mRNA in whole lungs, normalized by CD45 expression (n=4-5) (B-I). Data represent mean ± SEM; experimental groups are separately compared to the CC-LR cohort alone, where *p< 0.05 by unpaired t-test.


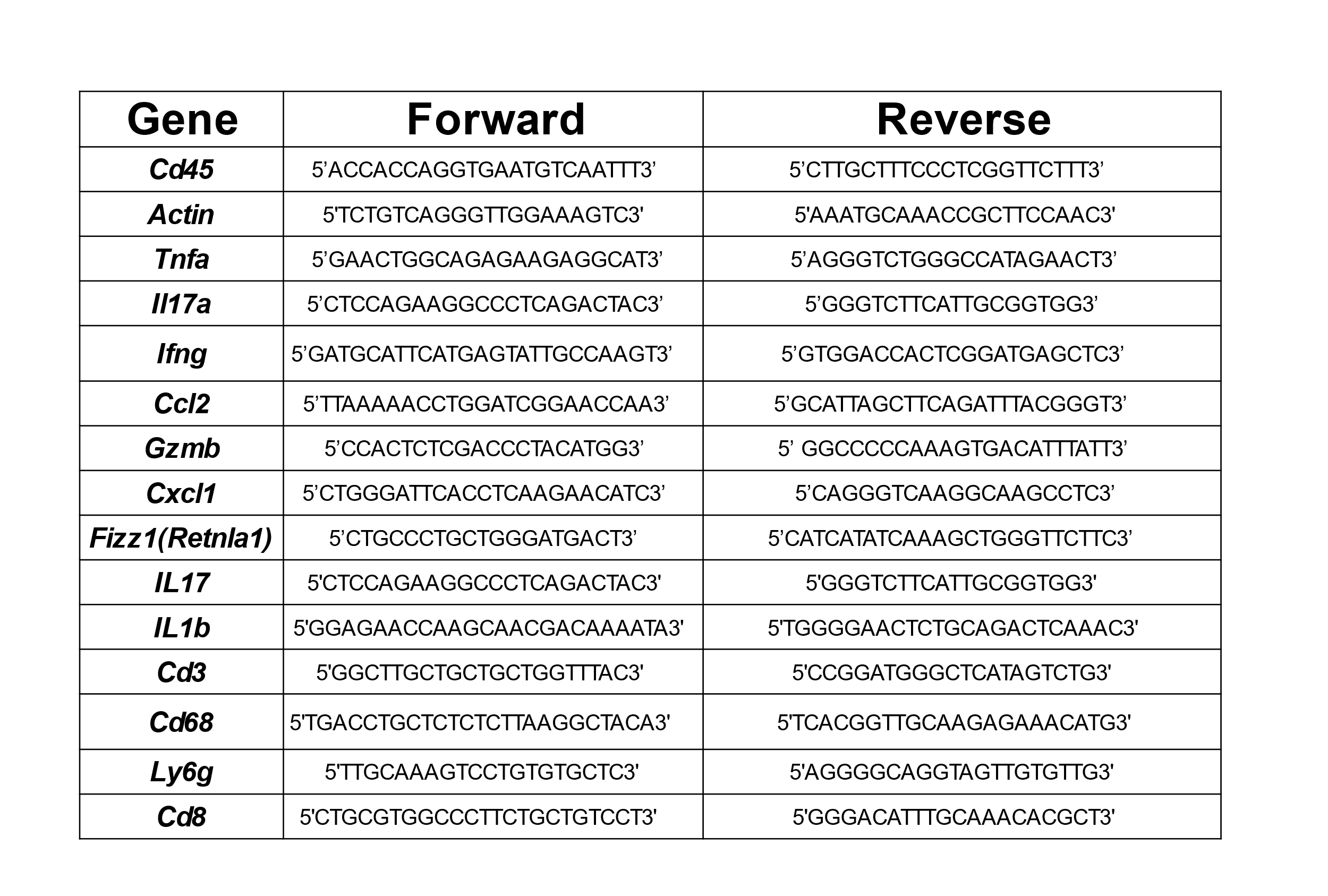


**Supplementary Table S9**. List of primers used in real-time qPCR
